# Genome Landscapes of Disease: Strategies to Predict the Phenotypic Consequences of Human Germline and Somatic Variation

**DOI:** 10.1371/journal.pcbi.1005043

**Published:** 2016-08-18

**Authors:** Rachel Karchin, Ruth Nussinov

**Affiliations:** 1 Department of Biomedical Engineering and Institute for Computational Medicine, Department of Oncology, Cancer Biology Program, Johns Hopkins Institutions, Baltimore, Maryland, United States of America; 2 Cancer and Inflammation Program, Leidos Biomedical Research, Inc., Frederick National Laboratory for Cancer Research, National Cancer Institute, Frederick, Maryland, United States of America; 3 Sackler Institute of Molecular Medicine, Department of Human Genetics and Molecular Medicine, Sackler School of Medicine, Tel Aviv University, Tel Aviv, Israel

Computational biology can marry disciplines to help solve some of the most pressing problems in medical research. When built on fundamental evolutionary, biological, and/or physics principles and provided with large quantities of diverse experimental data, computing power, and rigorous statistics tools, efficient and effective computational strategies can help unlock the secrets of the genome to cure disease. Computational biology has undertaken this challenge, spearheading efforts to decode the genetic blueprints encrypted in the human germline and in somatic variations to decipher complex phenotypic consequences. It devises new and creative approaches to sift through the immense—and rapidly growing—assembled human genetic material, its products, the proteome and the metabolome, and their possible clinical ramifications.

The cell is the basic unit of life; the nucleus houses the genetic material that is believed to have provided a distinct advantage to the evolving cell. The organization of the genome varies; it depends on cell type, stage of development, differentiation, disease status, and more. The higher-order spatial and temporal organization of genomes—which itself is a function of conditions and environment—is a driver of biological function in differentiation, development, and disease. Studies of genomics, epigenetics, big data analysis, imaging, and clinical cell and molecular biology all benefit from rigorous computational biology analyses and modeling. They profit from the testable hypotheses that computational biology provides. These link the genetic material to the physiological cell state; however, in-depth understanding of—and predicting—the phenotypic relationship to genome landscapes also requires effective algorithms to unravel the outcomes of small- and large-scale alterations on DNA, RNA, and protein molecules.

Diseases can be viewed as perturbed states of molecular systems. They can be of different types, including single-gene (monogenic) diseases and multifactorial diseases, such as cancers, immune system diseases, neurodegenerative diseases, cardiovascular diseases, and metabolic diseases. They may involve genetic alterations or be more complex. They can also be infectious diseases where interacting molecular networks of both pathogens and humans are involved, with the pathogen protein subverting the cell’s machinery.

The rapid development of next-generation methods for whole-genome, whole-exome, and targeted sequencing, complemented by earlier microarray technologies, including comparative genomic hybridization and single nucleotide polymorphism (SNP) genotyping arrays, has generated an unprecedented amount of data for analysis. The unraveling of the more-frequent-than-expected scope of submicroscopic copy-number variations (CNVs) and their link to structural variation and disease has yielded discoveries of novel phenotype associations. It is now well known that CNVs, as well as SNPs, are responsible for human evolution, genetic diversity, and susceptibility to genomic disorders. Some genomic disorders result from structural changes of the human genome that convey traits or susceptibility to traits. Such rearrangements are believed to occur because of architectural features of the genome that abet genome instability. Genome-wide association studies have revealed common variation, and targeted sequencing has provided fine mapping of the genomic regions surrounding common variants. New methods have been developed to detect and assess rare variant associations, enhancing the understanding of the genetic architecture of disease.

The most common form of germline intra-species variation is the single nucleotide variant (SNV). Recent sequencing of whole human genomes has identified the number of SNVs in each individual to be in the range of 3–5 million. Although most SNVs are likely to be benign, some SNVs have a pathogenic effect and thus directly contribute to disease susceptibilities and drug sensitivities. Discovering these pathogenic SNVs is one of the main goals of modern genetics and genomics studies. Large-scale sequencing of cancer genomes has further uncovered thousands of somatic DNA alterations present in the tumor cells but not in the germline DNA of sequenced individuals. The implications of these alterations, which include SNVs, copy number changes, and structural rearrangements, on patient diagnosis, prognosis, and treatment regimens have become one of the central issues in 21st century cancer biology and medicine.

The number of genomic alterations discovered by next-generation sequencing methods is too large to be directly assessed by wet bench methods, such as cell culture experiments and animal models, or by statistical associations in case-control or family-based study designs. Computational biology is increasingly playing a role in prioritizing genomic alterations most likely to be pathogenic or of clinical relevance in cancer because computer algorithms can perform high-throughput analysis of large datasets. Computer models can also integrate genomic data with information about gene expression, methylation, regulatory and protein interaction networks, as well as clinical and imaging data.

There is continuous increase in the scale of genomic datasets as well as in the difficulty of identifying functionally and clinically relevant genetic variation. Among the many challenges posed to computational biology is to push the boundaries of what we might be able to learn from the genome alone while considering its cell/tissue environment, physiological state, and occurrence in disease. If significant strides are made and specific associations and linkages are identified, progress will be made toward sufficiently robust predictions with rewarding prognostic medical implications.

*PLOS Computational Biology* has assembled a *Focus Feature* on the genome landscapes of disease: strategies to predict the phenotypic consequences of human germline and somatic variation. International efforts to sequence large patient cohorts and healthy control populations are generating exponentially increasing amounts of genomic data. Without the ability to interpret the phenotypic consequences of germline and somatic variation, this data collection will have limited value. We hope that this *Focus Feature* will inform the readership of *PLOS Computational Biology* about current state-of-the-art of computational methods to predict these consequences, provide perspective on key successes and limitations in this area to date, and point to areas where progress is urgently needed.

The *Focus Feature* includes three papers. In the first, Yoo-Ah Kim, Dong-Yeon Cho, and Teresa Przytycka point to genotype-phenotype effects in cancer, which can be revealed via network approaches. The authors argue that cancer is a complex and heterogeneous disease and no two cancer cases are identical. Capturing the similarities as well as the differences is essential for a better understanding of the disease and its treatment. The authors review network-based approaches to cancer data analysis, focusing on their roles in modeling inter-tumor heterogeneity.

Jonas Reeb, Maximilian Hecht, Yannick Mahlich, Yana Bromberg, and Burkhard Rost describe predictions of molecular effects of sequence variants to bridge the gap from the micro level of molecular function to the systems/macro level of disease. David Masica and Rachel Karchin emphasize the importance of increasing the clinical relevance of in silico methods to predict pathogenic missense variants. They highlight the rapid accumulation of variants of unknown clinical significance, most of which cause amino acid substitutions (cSNVs). To interpret these variants, there is an urgent need to develop better in silico bioinformatic methods. They critically review the current state of the field, pointing to the progress and shortcomings in the development of bioinformatics missense variant classifiers. In particular, they advocate the increased use of endophenotypes, which are quantitative measurements that are correlated with phenotypes via shared genetic causes.

Computational biology can tackle cancer through analysis of massive quantities of data. These analyses may allow correlating the data with phenotypic outcomes. Voluminous compilations permit reaping robust statistical trends, which can be exploited for predictions. Computation can also contribute to cancer research by unraveling the mechanisms through which particular genetic or acquired aberrations actually work. Molecular structures can contribute greatly to understanding of disease mechanisms and the design and computational screening of drugs. By combining data from genomics and molecular structure, computational biology can enable interpretation of genetic and epigenetic data to tailor disease treatments to individual patients. Together with experiments, the physical sciences, genetics, statistical “big data,” and clinical analysis, computational biology can help to lay the foundation for new paradigms in the translational sciences. We look forward to the time when these tools can elucidate the basis of cancer and provide predictive blueprints and combinatorial drug therapies. However, the challenge facing us is daunting.

